# A New IRAK-M-Mediated Mechanism Implicated in the Anti-Inflammatory Effect of Nicotine via α7 Nicotinic Receptors in Human Macrophages

**DOI:** 10.1371/journal.pone.0108397

**Published:** 2014-09-26

**Authors:** Maria C. Maldifassi, Gema Atienza, Francisco Arnalich, Eduardo López-Collazo, Jose L. Cedillo, Carolina Martín-Sánchez, Anna Bordas, Jaime Renart, Carmen Montiel

**Affiliations:** 1 Departamento de Farmacología y Terapéutica, Facultad de Medicina, Universidad Autónoma de Madrid, IdiPAZ, Madrid, Spain; 2 Servicio de Medicina Interna, Hospital Universitario “La Paz” de Madrid, IdiPAZ, Madrid, Spain; 3 Laboratorio de Inmunología Tumoral, Hospital Universitario “La Paz” de Madrid, IdiPAZ, Madrid, Spain; 4 Instituto de Investigaciones Biomédicas “Alberto Sols”, Consejo Superior de Investigaciones Científicas-Universidad Autónoma de Madrid, IdiPAZ, Madrid, Spain; Taipei Medical University, Taiwan

## Abstract

Nicotine stimulation of α7 nicotinic acetylcholine receptor (α7 nAChR) powerfully inhibits pro-inflammatory cytokine production in lipopolysaccharide (LPS)-stimulated macrophages and in experimental models of endotoxemia. A signaling pathway downstream from the α7 nAChRs, which involves the collaboration of JAK2/STAT3 and NF-κB to interfere with signaling by Toll-like receptors (TLRs), has been implicated in this anti-inflammatory effect of nicotine. Here, we identifiy an alternative mechanism involving interleukin-1 receptor-associated kinase M (IRAK-M), a negative regulator of innate TLR-mediated immune responses. Our data show that nicotine up-regulates IRAK-M expression at the mRNA and protein level in human macrophages, and that this effect is secondary to α7 nAChR activation. By using selective inhibitors of different signaling molecules downstream from the receptor, we provide evidence that activation of STAT3, via either JAK2 and/or PI3K, through a single (JAK2/PI3K/STAT3) or two convergent cascades (JAK2/STAT3 and PI3K/STAT3), is necessary for nicotine-induced IRAK-M expression. Moreover, down-regulation of this expression by small interfering RNAs specific to the IRAK-M gene significantly reverses the anti-inflammatory effect of nicotine on LPS-induced TNF-α production. Interestingly, macrophages pre-exposed to nicotine exhibit higher IRAK-M levels and reduced TNF-α response to an additional LPS challenge, a behavior reminiscent of the ‘endotoxin tolerant’ phenotype identified in monocytes either pre-exposed to LPS or from immunocompromised septic patients. Since nicotine is a major component of tobacco smoke and increased IRAK-M expression has been considered one of the molecular determinants for the induction of the tolerant phenotype, our findings showing IRAK-M overexpression could partially explain the known influence of smoking on the onset and progression of inflammatory and infectious diseases.

## Introduction

Cigarette smoking stronggly influences the onset and clinical course of various pathologies through a complex set of actions affecting the body’s defense mechanisms that range from pro-inflammatory to immune-suppressive [1]–[4]. The specific mechanisms by which the individual components of cigarrette smoke affect the host’s inflammatory response are incompletely understood, so their study represents a first step toward establishing the most appropiate therapeutic strategies for the management of diseases in patients who are smokers.

Nicotine, a major component of tobacco smoke, has been shown to strongly modify the inflammatory response both *in vitro* and *in vivo* [5]–[8]. Its anti-inflammatory action, like that of the endogenous neurotransmitter ACh, is due to its binding to and activation of α7 nicotinic acetylcholine receptors (α7 nAChRs) on resident macrophages under the control of the ‘cholinergic anti-inflammatory pathway’ (CAP) [5], [6], [9], [10]. This anti-inflammatory pathway, first identified by the Tracey group, constitutes the efferent arm of a vagal nerve circuit directly linked to the immune system. The physiological activation of CAP is critical to ensure the appropriate magnitude of the inflammatory response to an infection or injury since it culminates in T cell release of ACh and the interaction of the neurotransmitter with α7 nAChRs on resident macrophages in the spleen, thereby inhibiting pro-inflammatory cytokine production by these macrophages [11], [12].

The discovery that CAP is a major modulator of immune cell function in a wide variety of inflammatory disease states [13], [14] has prompted the study of the downstream signaling from the α7 nAChRs involved in the ACh or nicotine anti-inflammatory effect. Available data reveal the involvement of signaling pathways that, directly or indirectly, lead to activation of the signal transducer and activator of transcription 3 (STAT3) via Janus-kinase 2 (JAK2) and/or to inhibition of the nuclear translocation of NF-κB, the first and last being central transcription factors for innate and adaptive immunity [7], [15], [16], [17]. Determining how STAT3 is involved in the anti-inflammatory effect mediated by α7 nAChRs is another challenge, although we know that activated STAT3 may act either directly or indirectly, depending on the type of activating stimulus. In the first case, phosphorylated STAT3 forms dimers that translocate into the nucleus, where they act as a negative regulator of proinflammatory cytokine expression [16], [17]. In the second case, STAT3 may converge and interfere with NF-κB activation in such a way that, upon becoming activated, a NF-κB p65 homodimer recruits and associates with STAT3 [18], [19].

Toll-like receptors (TLRs) are an essential receptor family for initiating activation of the innate immune response to various pathogens. To do this, these receptors recognize a variety of pathogen-associated molecular patterns (PAMPs), such as lipopolysaccharide (LPS) (endotoxin), which, in turn, activates TLR4 and triggers intracellular signaling pathways that lead to the production of pro-inflammatory cytokines [20], [21]. However, excessive immune response to TLR stimulation is detrimental to the host, even lethal, so this process must be tightly regulated by protective mechanisms that counteract excessive immune activity. An example of such mechanisms is ‘endotoxin tolerance’, a phenomenon reported in monocytes/macrophages that have either been pre-exposed to LPS or come from immunocompromised septic patients; these cells enter a transient refractory state of hyporesponsiveness to subsequent LPS challenge and are unable to produce pro-inflammatory cytokines at levels comparable to those seen during the first LPS exposure or before sepsis [22]–[24]. Other examples of protective mechanisms include a large number of signaling molecules whose expression can negatively regulate TLR signaling, and they thus play a pivotal role in controlling innate responses in immune cells [19], [22], [25]–[29]. These molecules include STAT3, suppressors of cytokine signaling 1 and 3 (SOCS-1, SOCS-3), phosphatidylinositol 3-kinase/threonine protein kinase B (PI3K/Akt), myeloid differentiation primary response gene 88 (MyD88), and interleukin-1 receptor-associated kinase-M (IRAK-M). Interestingly, some of these negative regulators of TLR signaling (STAT3, SOCS-3, PI3K and MyD88s) have also been implicated in the anti-inflammatory effect mediated by α7 nAChRs in immune cells [16], [30]–[32]. However, the participation of others, like IRAK-M, is yet to be evaluated.

IRAK-M, first characterized in humans [33], is a member of the IRAK family that, unlike other active members of this family (IRAK-1 and IRAK-4), lacks kinase activity. This inactive kinase (or pseudokinase) appears to be exclusively expressed in monocytes/macrophages of different species [33], [34], and its expression can be induced not only by TLR activation (e.g. with the TLR4 ligand, LPS), but also in response to endogenous or exogenous soluble factors (adiponectin or prostaglandin E2), as well as to cell surface or intracellular signaling molecules (such as PI3K) (see Ref. [29] and references therein). Once IRAK-M expression is induced by TLR ligands, it may act as a negative regulator of the TLR signaling pathway by preventing the dissociation of the active IRAKs (IRAK-1 and IRAK-4) from MyD88 and the subsequent formation of a complex with TNF receptor-associated factor 6 (TRAF6), thereby inhibiting downstream inflammatory signals and suppressing production of the pro-inflammatory mediators controlled by this signaling pathway [22], [24], [35]. Accordingly, IRAK-M-deficient macrophages produce higher levels of pro-inflammatory cytokines upon TLR/IL-1R stimulation. Based on the above findings, IRAK-M levels have been negatively associated with the magnitude of the inflammatory response in human sepsis, supporting a prominent role for IRAK-M in the regulation of immune homeostasis in infectious and non-infectious human diseases [23], [29], [36], [37]. Furthermore, up-regulation of IRAK-M and other negative TLR4 regulators, along with impaired activation of IRAK4, p38, and NF-κB, have been recognized as molecular determinants for the induction of ‘endotoxin tolerance’ in monocytes, which may have important pathophysiological implications *in vivo* [23], [36], [38].

We hypothesized that IRAK-M may contribute to the well-known anti-inflammatory effect of nicotine acting on α7 nAChRs in human macrophages on the basis of the above evidences, which are summarized as follows: 1) the overlapping expression of IRAK-M and α7 nAChRs in the same cell types (macrophages); 2) the decisive role of IRAK-M in negatively regulating the inflammation mediated by TLR signaling; and 3) the involvement of other negative regulators of TLR signaling in the anti-inflammatory effect mediated by α7 nAChRs in macrophages. The present study tested this hypothesis by analyzing the possible connection between nicotine and IRAK-M via α7 nAChRs in human macrophages and also by studying whether this molecular mechanism could be behind the anti-inflammatory and immunosuppresant effects of nicotine.

## Materials and Methods

### Reagents

Unless otherwise indicated, all products were purchased from SIGMA (Madrid, Spain). DMEM and RPMI media were from Gibco (Invitrogen, UK). PNU120596 and all of the inhibitors used in this study were purchased from Tocris Bioscience (Bristol, UK).

### Cell Isolation and Culture

Human PBMC were isolated as described elsewhere [23], [39]–[41] from blood buffy coat cells from healthy individual donors provided anonymously by the Blood Transfusion Center of the Comunidad de Madrid. Data from these blood samples were analyzed anonymously. Cells were collected, washed, resuspended in complete RPMI 1640 medium (GIBCO, Invitrogen) without serum and seeded onto 60 mm Petri dishes (2×10^7^ cells per dish), onto coverslips in 24-well culture plates (10^6^ cells per well), or onto 6-well culture plates (10^7^ cells per well). After 2 hours, the adherent monocyte-enriched cell population (about 10% of total PBMC) was washed with the medium and monocytes allowed to diferentiate into macrophages for 3, 8 or 12 days in the presence of M-CSF (2 ng.ml^−1^) and complete culture medium with 5% human serum in a 5% CO_2_ environment at 37°C following the protocol described by the Kevin J Tracey group [5], [6], [9]. Unless otherwise noted, most experimental protocols were done with human macrophages (MØ) differentiated for 10–12 days from monocytes and confirmed by CD14 and CD89 positive cell surface expression. Macrophages from the RAW 264.7 cell line (immortalized mouse leukemic monocyte macrophage cells, from American Type Culture Collection ATCC, Manassas, VA, USA) were maintained in RPMI 1640 medium supplemented with 10% FBS in a humidified incubator in a 5% CO_2_ environment at 37°C. Cells were subcultured before confluence, seeded on 60 mm Petri dishes, and then allowed to grow to 80% confluence on the dish before being subjected to different treatments.

### Protein Extraction and Western Blot Analysis

After being subjected to different treatments, MØ or RAW 264.7 cells were placed on ice and washed with cold PBS. Cells were lysed with lysis buffer [50 mM Tris-HCl, 150 mM NaCl, 1 mM EDTA, 1% w/w NP-40; pH 8.0] containing a complete protease inhibitor cocktail (Sigma-Aldrich, USA) and phosphatase inhibitors (5 mM sodium fluoride, 1 mM sodium orthovanadate and 5 mM beta-glycerophosphate) in the case of phosphorylation assays. Protein concentration was determined by a BCA assay kit (Pierce BCA Protein Assay kit, Thermo Fisher Scientific, USA), and gels loaded with equal amounts of protein per lane (30–40 µg protein/lane). Next, proteins were resolved by 10% SDS/PAGE gel electrophoresis, transferred to a PVDF membrane (Millipore), and analyzed by Western blot using the appropriate antibodies. The primary antibodies [rabbit anti-IRAK-M polyclonal antibody (Millipore) or goat anti-β-actin monoclonal antibody (Santa Cruz Biotechnology, CA, USA)] were diluted 1∶1000 in TTBS buffer, and incubated 1 hour at room temperature. For phosphorylation assays, blots were incubated overnight at 4°C with the following anti-phospho and non-phospho primary antibodies (dilution 1∶1000) from Cell Signaling (Beverly, MA, USA), unless otherwise indicated: rabbit monoclonal anti-pERK1/2 (Thr202/Tyr204); rabbit polyclonal anti-ERK (Millipore); rabbit polyclonal anti-pJAK2 (Tyr1007/1008); rabbit polyclonal anti-JAK2; rabbit monoclonal anti-pSTAT3 (Tyr705), rabbit polyclonal anti-STAT3; rabbit monoclonal anti-pAkt (Ser473); and rabbit polyclonal anti-Akt. Proteins were detected using the appropiate secondary (HRP)-conjugated antibody (from Jackson Immuno Research PA, USA, or Cell Signaling) and incubated for 1 hour at room temperature at the dilution recommended by the manufacturer. The resulting bands were detected using ECL Plus reagents (Amersham, GE Healthcare, UK) and quantified by densitometry using Image J software (National Institutes of Health, USA).

### Quantitative Real-Time PCR (qPCR) Analysis of Gene Expression

The level of IRAK-M mRNA expression in MØ was assessed by qPCR from reverse-transcribed total RNA previously isolated from the cells with the RNeasy Mini kit (Qiagen, Hilden, Germany) as described [39]. A SYBR green-based assay was used for amplicon detection on the ABI Prism 7500 device. The following primers were used: for IRAK-M, forward 5’-TTTGAATGCAGCCAGTCTGA and reverse 5’-GCATTGCTTATGGAGCCAAT; for β-2-Microglobulin (B2M), used as an internal control, forward 5’-TGCCTGCCGTGTGAACC-ATGT and reverse 5’-TGCGGCATCTTCAAACCTCCATGA. Analysis of the melting curves demonstrated that each pair of primers amplified a single product. qPCR for IRAK-M mRNA was performed in triplicate and later normalized to the expression of B2M mRNA. Relative gene expression values were calculated by the comparative 2^−ΔΔCt^ method using Sequence Detection System 1.2 software (Applied Biosystems).

### Flow Cytometry

Monocyte-enriched population seeded onto 60 mm Petri dishes was washed with PBS and detached from the plate using 5 mM EDTA/PBS solution. The cell suspension was centrifuged and the resulting pellet incubated for 20 minutes at 4°C with a 2 mM EDTA/0.5% FBS/PBS solution containing the mouse anti-human CD89-FITC (AbD SEROTEC, Oxford, UK) and the mouse anti-human CD14-APC (Miltenyi Biotec, Bergisch Gladbeach, Germany). In another set of experiments, the pellet obtained as above, but starting with cell suspensions of human monocytes at various stages of differentiation to MØ (3, 8 and 12 days), were incubated for 20 minutes at 4°C with a 2 mM EDTA/0.5% FBS/PBS solution containing a combined mixture of the specific labelled toxin for the α7 nAChR, αBgtx-FITC (3 µg. ml^−1^, Sigma), and mouse anti-human CD14-APC. After staining, cells were washed and resuspended in 2 mM EDTA/PBS solution and processed on a BD FACSCanto II flow cytometer equipped with BD FACSDiva software.

### Confocal Microscopy Analysis of Receptor Expression

The native expression level of functional α7 nAChRs in MØ was also analyzed by confocal microscopy using αBgtx-FITC. Cells seeded on coverslips were incubated with the labeled toxin (3 µg. ml^−1^) as previously described [39]. After several rinses, MØ were fixed, washed, mounted and visualized with the Leica TCS SP2 Spectral confocal laser scanning microscope. All images were captured with the Leica Confocal Software using the same adjustments of laser intensity and photomultiplier sensitivity as described elsewhere [39], [42], [43].

### Gene Silencing by Transfection of Stealth RNA Interference (siRNA)

Two different non-overlapping Stealth™ RNAi duplexes (siRNA-1 and siRNA-2) designed for silencing the human IRAK-M gene (NM_007199.2) and the corresponding negative Stealth™ RNAi duplex control (siRNA control; Medium GC Duplex) were purchased from Invitrogen (Life Technologies, Paisley, UK). The IRAK-M siRNA-1 and IRAK-M siRNA-2 sequences were designed to target base pairs 1336–1360 (sense strand, 5’-CGG AAU UUC UCU GCC AAG CUC UUC U-3’; antisense strand, 5’-AGA AGA GCU UGG CAG AGA AAU UCC G-3′) and base pairs 1487–1511 (sense strand, 5’-CCU UCA GGU GUC CUU CUC UAU U-3’; antisense strand, 5’-AAU AGA CGA GAA GGA CAC CUG AAG G-3’) of the IRAK-M gene. Before transfection, human PBMC were seeded in 6-well culture plates and the adherent monocyte-enriched population allowed to diferentiate into MØ in complete RPMI 1640 medium (plus 5% v/v human serum and 2 ng.ml^−1^ M-CSF) as described before. Silencing of the IRAK-M gene was performed according to the protocol previously described for human macrophages [44]. Briefly, cells were incubated at 37°C for 6 h with a transfection master mix containing fresh serum-free OptiMEM medium (Invitrogen, Life Technologies), the siRNA-1 or siRNA-2 (or the negative siRNA control) at a final concentration of 1 nM in the well, and INTERFERin (Polyplus, Illkirch, France). Then, the above master mix was replaced overnight by serum-free RPMI 1640 medium. After this period, MØ were exposed to various treatments specified in the Results section and then the IRAK-M expression knock-down was analyzed in the protein extract from the cell lysate using Western blot and the TNF-α concentration in the cell supernatants using ELISA.

### Tolerance Induction

A Monocyte-enriched population was cultured in 6-well culture plates and allowed to diferentiate into MØ in complete RPMI 1640 medium. The cells were divided into four groups (5 wells/group). Group 1 was maintained in culture medium alone throughout the experiment (48 h). Group 2 was pre-incubated in culture medium for 8 h, washed three times with fresh medium and maintained in culture for 16 h, and then stimulated with LPS for 24 h. Groups 3 and 4 were preincubated with LPS or nicotine for 8 h respectively, washed as in group 2, and stimulated with LPS for 24 h. Upon completion of the protocol, we proceeded to perform Western blot to analyze IRAK-M expression in the cell extract, and ran an ELISA analysis to detect TNF-α in cell supernatants from all four groups.

### Cytokine Detection by Elisa Analysis

At the end of the cell treatments, supernatants were harvested and stored at −20°C. Levels of TNF-α in the culture supernatants were determined using a commercial ELISA kit (Human TNF-α ELISA Development Kit from PeproTech EC Ltd., London, UK) according to the manufacturer’s instructions.

### Statistical Analysis

All of the data are presented as mean values ± SEM from at least three independent experiments. One-way analysis of variance (ANOVA) was used for multiple comparisons between the groups followed by a specific post-hoc test (Bonferroni) to analyze the differences. Student’s *t*-test was used when only 2 groups were compared. A p≤0.05 was considered statistically significant.

## Results

### Nicotine Induces IRAK-M expression in Human and Mouse Macrophages

**Figure 1:**
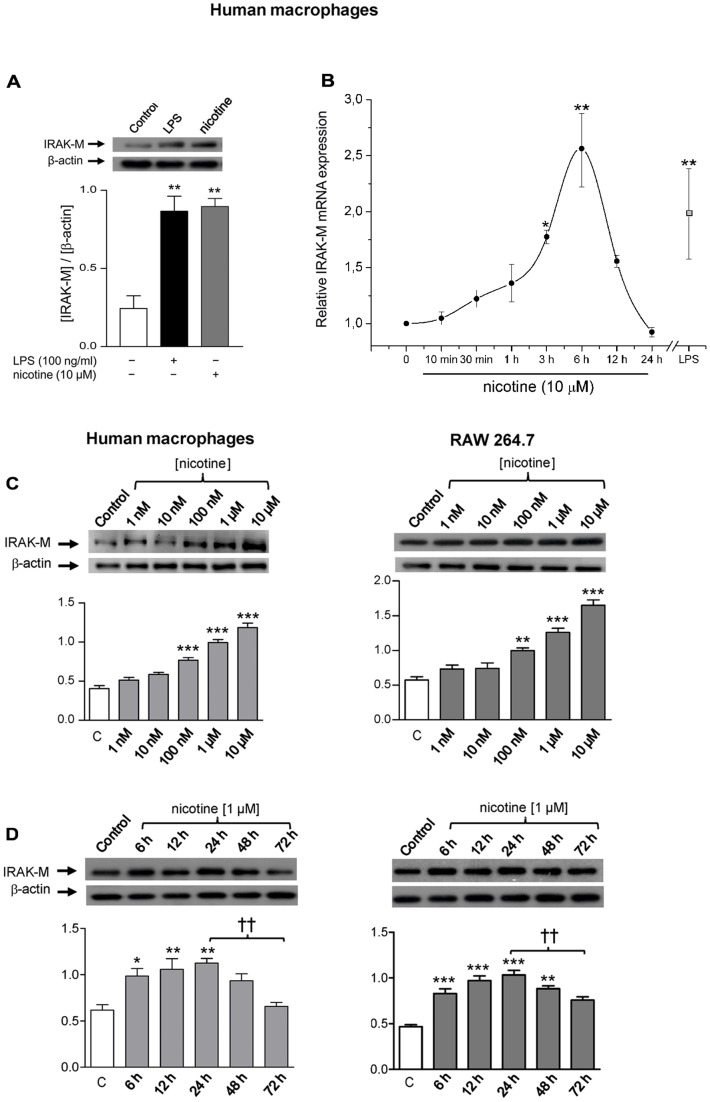
Nicotine induces IRAK-M expression in human and mouse macrophages. (A) Western blot analysis of IRAK-M levels in human macrophages (MØ) stimulated with nicotine or LPS (24 h). (B) Time-course for qPCR analysis of IRAK-M gene expression in nicotine-stimulated MØ; the square at the right shows the effect of LPS (100 ng.ml^−1^, 6 h); data represent mean ± SEM of the number of times that mRNA expression is increased by the stimulus as compared to non-stimulated cells from the same culture (n = 5 different cultures). (C, D) Analysis of IRAK-M protein levels in MØ and mouse macrophages (RAW 264.7) exposed to increasing concentrations of nicotine (24 h) or incubated with 1 µM nicotine for the indicated periods. The bars at the bottom of panels *A*, *C* and *D* show pooled results (mean ± SEM) from 5–6 different batches of cells subjected to the indicated treatment; a typical immunoblot from these experiments is shown in the upper part of these panels. *p≤0.05, **p≤0.01 and ***p≤0.001 comparing stimulated with non-stimulated cells from the same culture (control). ^††^p≤0.01 after comparing the indicated bars.

Nicotine attenuates LPS-induced pro-inflammatory cytokine production in macrophages while IRAK-M is a negative regulator of innate TLR/IL-1R-mediated immune responses in the same cell type. To assess whether there is any relationship between nicotine and this negative inflammation regulator, we used immunoblotting and qPCR assays to measure the nicotine effect on IRAK-M expression in human macrophages (MØ) differentiated from monocytes. IRAK-M protein levels were significantly higher in cells stimulated with nicotine (24 h) compared to non-stimulated cells from the same culture (Figure 1A). In fact, nicotine caused a ∼3.5-fold increase in IRAK-M levels, an effect similar to that obtained when incubating MØ with LPS for a similar period of time (Figure 1A). To analyze whether the up-regulation of IRAK-M elicited by nicotine was the result of increased gene transcription, we performed qPCR experiments to determine the time-course of IRAK-M mRNA levels in MØ stimulated with nicotine for different periods (Figure 1B). Non-stimulated cells or cells stimulated with LPS (100 ng.ml^−1^, 6 h) from the same batch were used as negative and positive controls, respectively. We performed qPCR with total RNA isolated from MØ using B2M as an internal control and the primers listed in the “Material and Methods” section. Our results show that nicotine up-regulates IRAK-M mRNA expression, with levels peaking at 6 h and then declining to baseline levels at 24 h of incubation with the drug.

The threshold concentration of nicotine and its kinetic effect on IRAK-M expression in MØ could be reproduced in macrophages from a different species, the mouse RAW 264.7 cell line. Figure 1C shows the Western blot analysis of results obtained in each cell type incubated with varying concentrations of nicotine applied for 24 h. In both types of macrophages, the nicotine effect is seen at concentrations above 100 nM and then increases in parallel to the concentration of the agonist. Figure 1D shows the Western blot analysis for the time-course of the nicotine (1 µM, at the indicated times) effect on IRAK-M expression in MØ and RAW 264.7 cells. Results reveal that the effect begins to be significant at 6 hours, reaches a maximum at 24 h and declines thereafter. For this reason, a stimulation period of 24 h was selected for subsequent experiments.

### The α7 nAChR Subtype is involved in the Nicotine-induction of IRAK-M Expression in MØ

**Figure 2:**
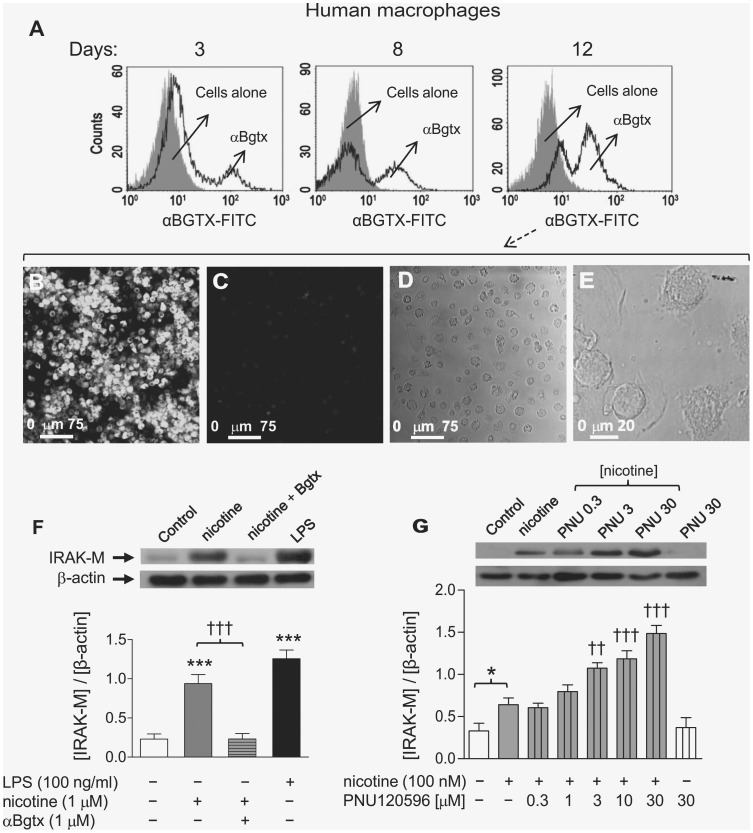
The α7 nAChR subtype is involved in the nicotine induction of IRAK-M expression in human macrophages. Expression of α7 nAChRs in human monocytes at different stages of differentiation into MØ was assessed by FACS (A) or confocal microscopy (B, C) using the selective toxin αBgtx-FITC (3 µg.ml^−1^). FACS analysis shows the expression patterns of αBgtx binding sites in CD14^+^ cells (black-lined histograms) and in control cells not-incubated with anti-CD14^+^ or αBgtx-FITC at the same day of culture (cells alone, gray-shaded histograms); results were reproduced in 4 independent experiments. Typical confocal images from the same culture (n = 4) corresponding to 12-day-old differentiated MØ stained with αBgtx-FITC with (C) or without (B) pre-incubation with a high concentration of nicotine (500 µM). (D) Transmitted light images of the cells in the same field as C, and (E) from a different field of the same culture at a higher magnification to reveal the morphological changes of the cells with differentiation. (F, G) Overexpression of IRAK-M protein induced by nicotine in differentiated MØ is completely abolished by αBgtx and potentiated by PNU120596; top panels show two typical immunoblots; the bottom panels show pooled results (mean ± SEM) obtained in 5–7 batches of cells assayed for each condition. *p≤0.05 and ***p≤0.001 after comparing nicotine- or LPS-stimulated with non-stimulated cells from the same culture. ^††^p≤0.01 and ^†††^p≤0.001 after comparison of nicotine-stimulated cells in the presence or absence of αBgtx or PNU120596.

FACS analysis of a representative culture sampling (n = 12) of all the cultures used in this study (n = 56) reveals that most of the adherent cells isolated from buffy coat blood from healthy volunteers were CD14^+^ (92.4±2.1%) and CD89^+^ (93.2±2.4%) cells (data not shown). This type of analysis performed on CD14^+^ cells labeled with αBgtx-FITC makes it possible to assess the expression level of α7 nAChRs in monocytes at different stages of their differentiation into macrophages (3, 8 and 12 days). Figure 2A shows that receptor expression is easily detectable on day 8 of differentiation and continues increasing until days 10–12. Thus, all experiments for the present study were performed in MØ differentiated for the longest time period. The expression of α7 nAChRs in MØ was also confirmed by confocal microscopy in cells immunostained with αBgtx-FITC, as has been reported previously in our laboratory [39]. Figure 2B shows a confocal image obtained in a typical culture of MØ differentiated for 12 days and stained with the labeled toxin. Receptor labeling with αBgtx-FITC is specific since it was abolished when macrophages from the same batch were preincubated with unlabeled 1 µM αBgtx (not shown) or with 500 µM nicotine (Figure 2C). Figure 2D shows a transmitted light image of the cells in the same field as Figure 2C, and Figure 2E of a different field from the same culture at a higher magnification to visualize morphological changes of the monocyte-derived macrophages with differentiation.

Once the optimal differentiation period to obtain the peak α7 nAChR expression had been established in MØ, we studied whether this receptor subtype was implicated or not in the nicotine effect on IRAK-M in these cells. To this purpose we evaluated the effect of αBgtx and PNU120596 on the nicotine effect; the former is a specific and potent inhibitor of α7 nAChR while the latter is a positive allosteric modulator of this receptor subtype. For these experiments, cells were incubated with αBgtx (30 min) or PNU120596 (10 min) before and during their nicotine incubation (24 h). Figure 2F shows that αBgtx completely abolished the nicotine effect on IRAK-M expression without affecting the LPS effect on that expression (not shown). Conversely, PNU120596 produced a dose-dependent increase in the nicotine effect, although the drug had no effect by itself, even at 30 µM, the highest concentration assayed in these experiments (Figure 2G). It should be noted that the concentration of nicotine used in the PNU120596 experiment was lower (100 nM) than the one used in previous experiments to make it easier to distinguish the enhancing effect of the positive allosteric modulator on the α7 nAChR-mediated signal.

### Signaling Pathways Connecting α7 nAChRs with IRAK-M in MØ

**Figure 3:**
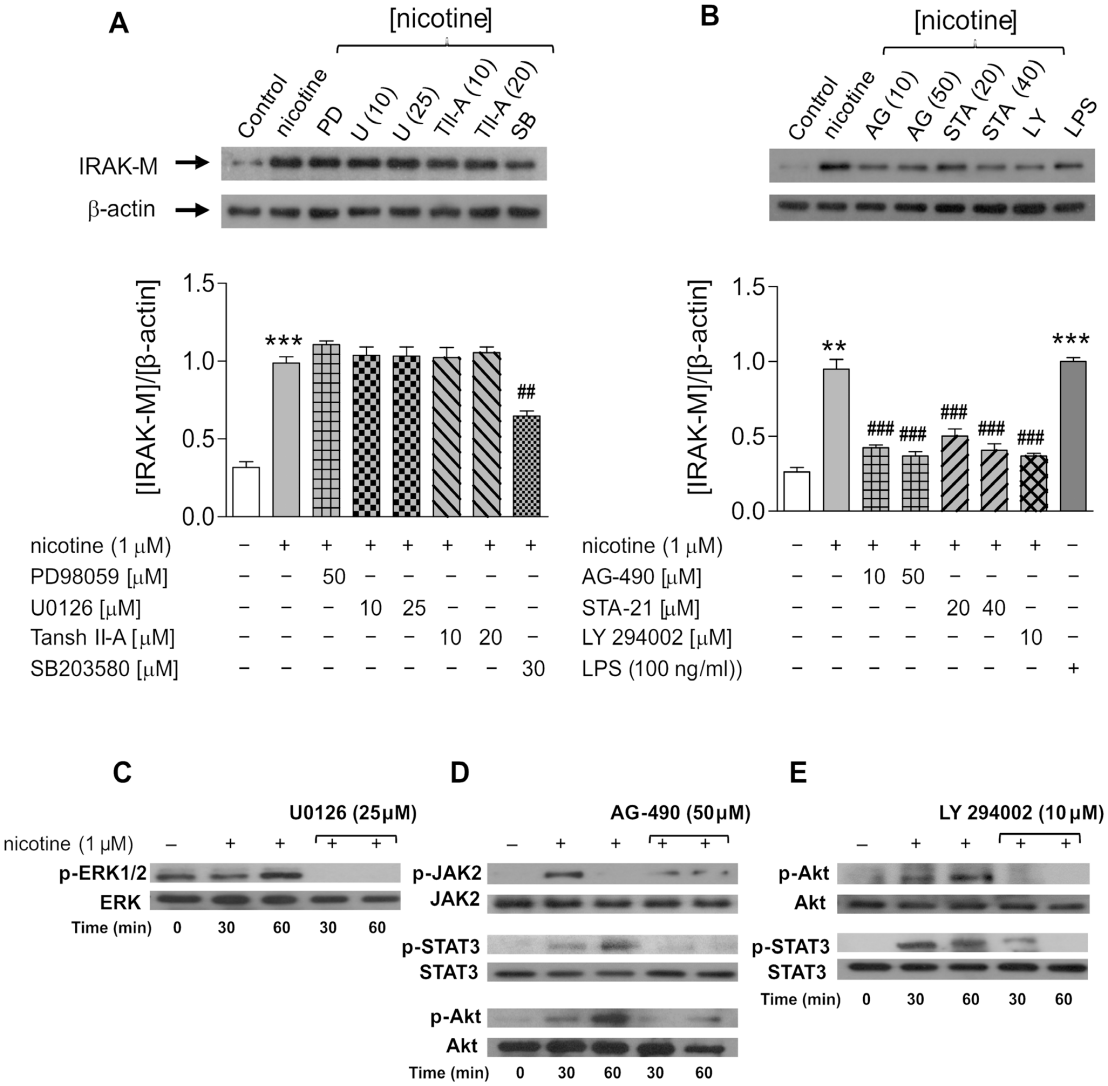
Pharmacological interference of signaling pathways that could connect α7 nAChRs with IRAK-M in human macrophages. The effects of selective inhibitors of different kinases [PD98059 (MEK1), U0126 (ERK1/2), SB203580 (p38^MAPK^), AG-490 (JAK2) and LY 294002 (PI3K)] and transcription factors [Tanshinone II-A (AP-1) and STA-21 (STAT3)] on nicotine-induced IRAK-M expression (A, B) or on nicotine-mediated phosphorylation of signaling molecules downstream from α7 nAChRs (C–E) were analyzed by Western blot using the appropriate antibodies (see Methods section). The target signaling molecules downstream from the receptor were selected based on literature data (see References in the corresponding Results section). The inhibitors, at the indicated concentrations, were added to the cell culture 30 min before and during the nicotine stimulation (24 hours for IRAK-M expression, and 30 or 60 min for phosphorylation assays). (A, B) The upper part shows two typical immunoblots with all the inhibitors assayed. The bars show pooled results (mean ± SEM) from 6–8 batches of cells assayed for each condition. **p≤0.01 and ***p≤0.001 after comparing nicotine- or LPS-stimulated with non-stimulated cells from the same culture. ^##^ p≤0.01 and ^###^p≤0.001 after comparing the effect of the corresponding inhibitor on the up-regulated IRAK-M expression elicited by nicotine. (C–E) Immunoblots evaluating the activation of different routes triggered by nicotine in MØ and the effect of blockade of the first kinase in each route (ERK1/2, JAK2 or PI3K) with its respective selective inhibitor (U0126, AG-490 and LY 294002). PVDF membranes with electrotransfered proteins were probed for phospho-ERK1/2, phospho-JAK2, phospho-STAT3 and phospho-Akt antibodies, using the corresponding non-phospho antibodies (ERK, JAK2, STAT3 and Akt) as controls. Blots are representative of three independent experiments.

In order to ascertain the possible signaling pathways involved in the nicotine-mediated up-regulation of IRAK-M via α7 nAChRs in MØ, we took advantage of the availability of selective inhibitors of various kinases (PD98059 for MEK1; U0126 for ERK1/2; SB203580 for p38^MAPK^; AG-490 for JAK2 and LY 294002 for PI3K) and transcription factors (Tanshinone II-A for AP-1 and STA-21 for STAT3) that have been recognized as signaling molecules downstream from the α7 nAChRs in different cell types, including macrophages and neurons [16], [17], [31], [45]–[53]. In these experiments, the effects of the above inhibitors on nicotine-induced IRAK-M expression (Figure 3A–B) or on nicotine-mediated phosphorylation of signaling molecules downstream from the α7 nAChR (Figures 3C–E) were assessed in MØ by Western blot. For IRAK-M expression analysis, the inhibitors were preincubated 30 min before and during the nicotine stimulus (1 µM, 24 h), at the concentrations indicated in the Figure. MØ from the same buffy coat culture either non-stimulated with nicotine or incubated with LPS (100 ng.ml^−1^, 24 h) were used as respective negative and positive controls for IRAK-M expression. Immunoblotting revealed that, with the exception of the inhibition of p38^MAPK^ leading to a partial, yet significant, reduction in the nicotine-dependent up-regulation of IRAK-M, the inhibition of MEK, ERK1/2 or AP-1 left the nicotine effect untouched, even at the highest concentration of the inhibitor used (Figure 3A). Conversely, inhibition of JAK2, STAT3 or PI3K, caused a greater-than-80% inhibition of the nicotine effect on protein expression, even at the lowest inhibitor concentration used (Figure 3B).

The finding that JAK2, STAT3 and PI3K contribute equally to nicotine-induced IRAK-M overexpression (Figure 3B) suggests that these three signaling molecules may be chained or converge in a common signaling pathway leading to the induction of IRAK-M. To test this hypothesis we focused our attention on those signaling routes that, according to previous studies (for more detail, see references in the corresponding section of Discussion), provide connection for the above signaling molecules: activated JAK2 can phosphorylate either STAT3 or PI3K/Akt and activated PI3K can phosphorylate STAT3. Once we had established the signaling pathways of interest, we used Western blot to explore whether the specific blockade of the first signaling molecule in each cascade affected the nicotine-mediated phosphorylation of signaling molecules downstream. The inhibitors were preincubated 30 min before and during the nicotine stimulus (1 µM, 30 or 60 min), at the concentrations indicated at the bottom of Figure 3 (panels D–E). The results show that the inhibition of JAK2 (AG-490) reduced the phosphorylation of both STAT3 and PI3K/Akt induced by nicotine (panel D); similarly, inhibition of PI3K (LY 294002) also interrupts the nicotine-mediated phosphorylation of STAT3 (panel E). Moreover, although we have found that 60 min of incubation with nicotine activates ERK1/2 in MØ (Figure 3C), this route did not contribute to the effect of nicotine on IRAK-M expression, as can be inferred from the lack of effect on expression (Figure 3A) by the selective inhibitor of the kinase (U0126).

### Specific IRAK-M Gene Silencing Suppresses the Anti-inflammatory Effect of Nicotine on TNF-α Production in LPS-stimulated MØ

**Figure 4:**
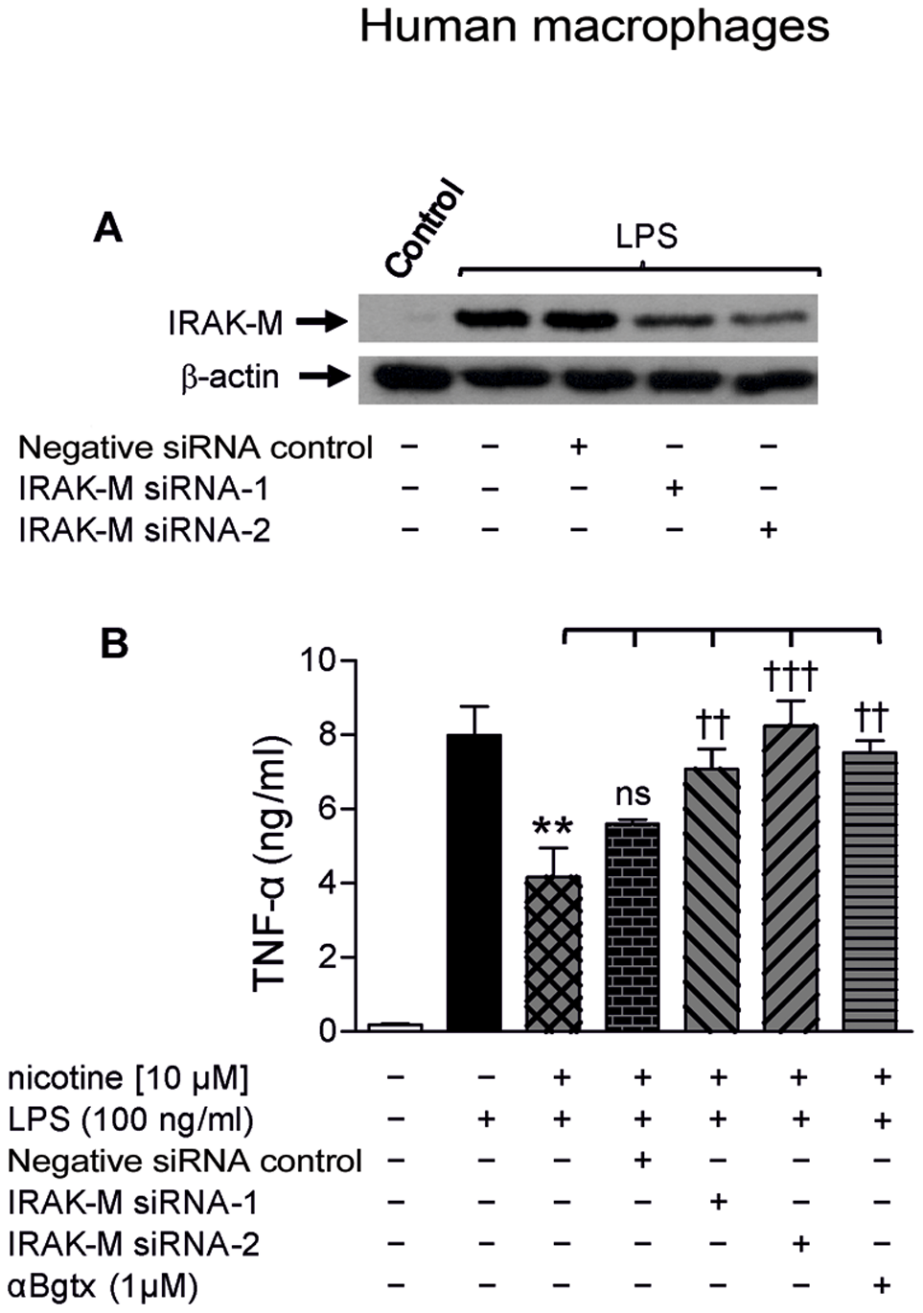
Knockdown of IRAK-M suppressed the anti-inflammatory effect of nicotine in LPS-stimulated human macrophages. (A) Typical immunoblot showing the knockdown of IRAK-M expression induced by LPS (100 ng.ml^−1^; 18 h) in MØ transfected with two non-overlapping siRNA duplexes (siRNA-1 and siRNA-2) specific to the IRAK-M gene, but not in cells transfected with the negative siRNA control. (B) ELISA assay of the anti-inflammatory effect of nicotine on TNF-α production in LPS-stimulated MØ as a function of the silencing of IRAK-M gene expression. The bars show pooled results (mean ± SEM) from 5 batches of cells assayed for each condition. **p≤0.01 after comparing the cytokine production in LPS-stimulated MØ from the same culture in the absence or presence of nicotine. ^††^p≤0.01 and ^†††^p≤0.001 after comparing the reversal of the anti-inflammatory effect of nicotine as a function of the experimental condition being tested. As expected, the reversal was complete after pre-incubation with αBgtx.

In order to test whether up-regulated IRAK-M gene expression is involved in the anti-inflammatory effect of nicotine, we proceeded to evaluate the nicotine effect on cytokine production in MØ stimulated with LPS (100 ng.ml^−1^; 18 h) after silencing IRAK-M gene expression with two different non-overlapping siRNA duplexes (siRNA-1 and siRNA-2). Nicotine pre-incubation of cells was started 3 h before and continued during the LPS stimulation. As illustrated in Figure 4A, a typical Western blot from five independent experiments, the mean ± SEM values of IRAK-M expression dropped by 44.4±3.3% and 66.0±2.2% in LPS-activated cells transfected with siRNA-1 or siRNA-2 respectively, compared to non-transfected cells or cells transfected with the negative siRNA control. Figure 4B shows, in parallel with the above Western blots, the effect of nicotine on TNF-α production in LPS-simulated MØ as a function of silencing of IRAK-M gene expression. Results reveal that the anti-inflammatory effect of nicotine on the TNF-α production in simulated MØ was significantly reversed by cell transfection with siRNA-1 or siRNA-2, but not with the negative siRNA control. As expected, αBgtx, placed in the incubation medium 45 min before and during the nicotine treatment, also produced a significant reversal of the nicotine anti-inflammatory effect in LPS-simulated cells.

### Pre-incubation of MØ with Nicotine Enhances IRAK-M Expression and Inhibits TNF-α Production in Response to LPS

**Figure 5:**
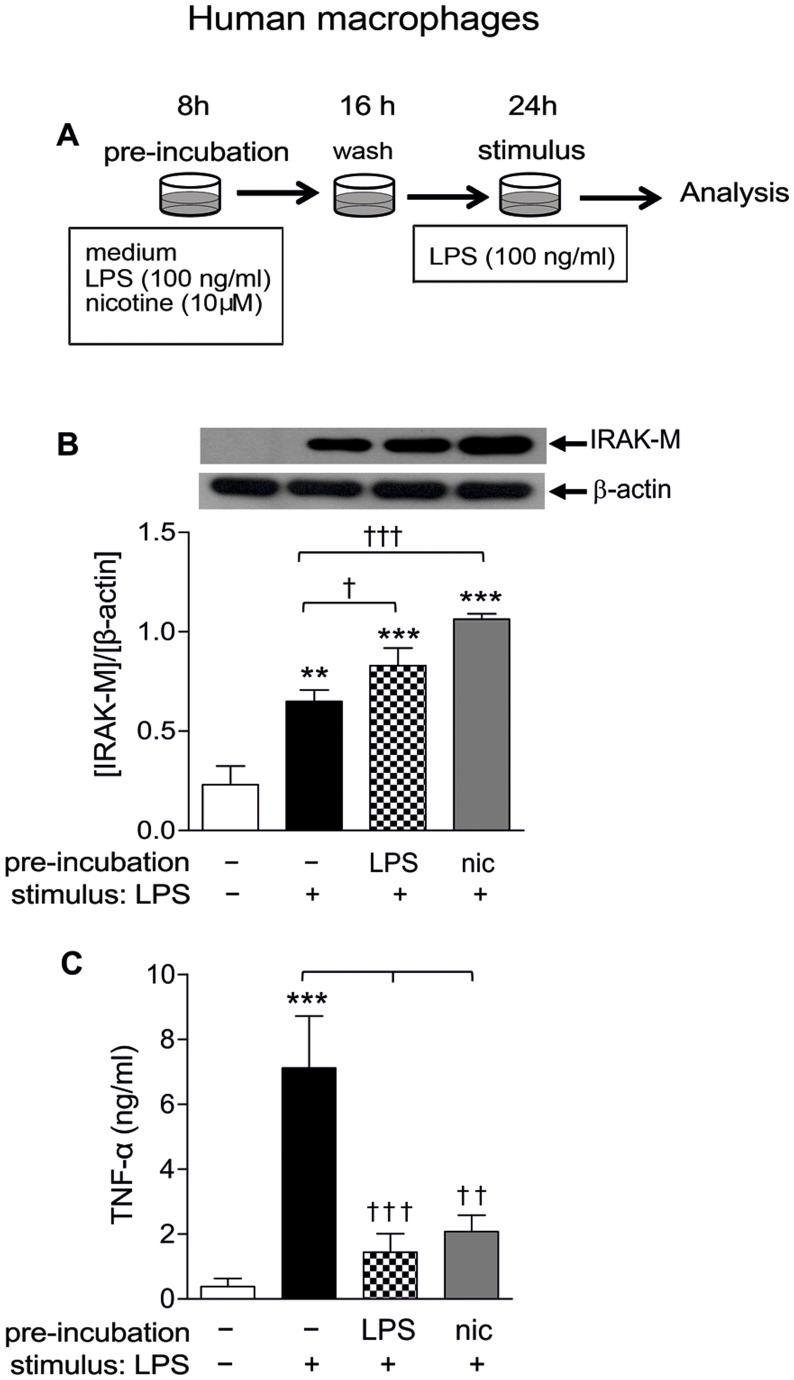
Nicotinic pretreatment of human macrophages raises IRAK-M expression and reduces TNF-α response to LPS, as in endotoxin tolerance. (A) Scheme of the experimental design used to test whether nicotine pretreatment can, like LPS, induce a refractory state in MØ, leading to a reduced inflammatory response to subsequent LPS challenge. (B) Typical immunoblot showing the IRAK-M expression in three groups of LPS-stimulated MØ preincubated with culture medium, LPS or nicotine; a fourth group of cells from the same batch maintained in culture medium throughout the experiment (48 h) was used as control (white bar). (C) ELISA analysis of TNF-α levels in MØ supernants from the four groups listed in *B*. The bars show pooled results (mean ± SEM) of 5 different cultures assayed for each condition. **p≤0.01 and ***p≤0.001 after comparing any of the three groups of LPS-stimulated MØ with non-stimulated cells from the same culture (control group).^ †^p≤0.05, ^††^p≤0.01 and ^†††^p≤0.001 after comparing the indicated bars with LPS-stimulated MØ preincubated with culture medium.

Monocytes/macrophages previously exposed to LPS develop ‘endotoxin tolerance’, which makes the cells hypo-responsive to subsequent LPS exposure. Up-regulation of IRAK-M has been identified as one of the molecular determinants implicated in the induction of this process. Since our study shows that nicotine reproduces the effect of LPS on IRAK-M expression in MØ, we wanted to investigate whether the cholinergic agonist can, like LPS, induce a refractory state in the cells, leading to a reduced inflammatory response to subsequent LPS challenge. The results in Figure 5 indicate that this is the case; the figure also includes the various experimental conditions tested (Figure 5A). By respectively doing Western blot and ELISA assays in the MØ extract and supernatants, we proceeded to simultaneously determine IRAK-M expression and TNF-α levels in each experimental condition. This experimental design was repeated with five different cultures. The results show that MØ pre-exposed to either nicotine or LPS present a significantly enhanced IRAK-M expression and decreased TNF-α response to a subsequent stimulation with LPS in comparison to cells that have not been pre-exposed to either nicotine or LPS (Figure 5B–C).

## Discussion

This study provides a novel mechanistic explanation for the anti-inflammatory effect of nicotine downstream from α7 nAChRs in human macrophages: IRAK-M gene expression is upregulated by nicotine, and this has an anti-inflammatory effect. In addition, our study also reveals that exposure to nicotine, as previously described with LPS exposure, reprograms macrophages into a refractory and hyporesponsive state toward additional TLR stimulations that might compromise the normal immune response.

In our study, nicotine induces overexpression of IRAK-M protein in MØ to levels similar to the response to LPS, which is used as the reference stimulus for up-regulation of this pseudo-kinase (Figure 1A); furthermore, this nicotine effect is exerted at the transcriptional level (Figure 1B). The kinetics of mRNA expression elicited by nicotine mimic those previously reported for LPS in human monocytes, blood leukocytes, or mice macrophages, in which maximum expression is reached after 3–6 h of stimulation [22], [23], [40].

It is worth noting that the IRAK-M up-regulation induced by nicotine in MØ also occurs in macrophages from a different species, the mouse (Figure 1C–D). In fact, no significant differences in relation to the threshold concentration of nicotine or its time-course for stimulating IRAK-M expression were found between macrophages from either species. Furthermore, the time-course of the nicotine effect on protein expression in the two types of macrophage (Figure 1D) is very similar to that obtained in murine macrophages using adiponectin or LPS as stimuli [54]. Taken together, the above results indicate that induction of IRAK-M by nicotine is a reproducible and generalized process in macrophages, regardless of the species tested.

Vagus nerve stimulation, as well as exposure to ACh or nicotine, inhibits the production of TNF-α, IL-1β, IL-6, IL-8, high-mobility group box 1 (HMGB1), PGE_2_ and MIP-1α in LPS-activated mouse or human macrophages [5], [6], [9]. Some of these studies have also revealed that α7 nAChRs are specifically responsible for these anti-inflammatory effects. However, the α4β2 nAChR subtype expressed in murine alveolar macrophages has also been implicated in the nicotine-induced suppression of alveolar macrophage activation by *Legionella pneumophila*, a causative agent for pneumonia [55]. Thus, nicotine treatment of these cells after infection with *L. pneumophila* significantly and selectively down-regulated the production of TNF-α, IL-6 and IL-12; moreover, this anti-inflammatory effect could be completely blocked by the non-selective antagonist for nAChRs, *d*-tubocurarine, but not by αBgtx.

The above background suggests that at least two nAChR subtypes may be involved in the nicotine induction of IRAK-M expression in macrophages, although our data (Figure 2F–G) clearly indicate that in MØ only α7 nAChRs are actually involved. Thus, the blockade of this receptor subtype with the selective antagonist (αBgtx) completely abolished the nicotine effect on IRAK-M, while the positive allosteric modulator of α7 nAChRs [56], PNU120596, caused a large and significant concentration-dependent potentiation of the nicotine effect. It should be noted that the MØ used in our experiments (differentiated from monocytes for 10–12 days) abundantly express α7 nAChRs, as demonstrated by the FACS results and confocal microscopy images using αBgtx-FITC for receptor labeling (Figure 2A–B).

Although α7 nAChRs may interact with more than 50 different proteins to trigger multiple intracellular signals [16], [17], [31], [45]–[53], [57], our results obtained with selective inhibitors for different signaling molecules (Figure 3A–B) limit the number of possible pathways involved in the nicotine-mediated effect on IRAK-M expression to two kinases (JAK2 and PI3K) and one transcription factor (STAT3). This finding agrees with published observations [16], [31], [52] showing that these three components in the downstream signaling pathway from α7 nAChR are involved in most of the pathophysiological responses associated to this receptor subtype. Our study also shows that pharmacologically interfering in the MAPK pathway at different levels leaves the nicotine effect on IRAK-M unaltered (Figure 3A), except for p38^MAPK^ inhibition using SB203580, which partially and significantly blocks the IRAK-M up-regulation induced by nicotine. The contribution of p38^MAPK^ to the nicotine effect may be the result of the ability of this member of the MAPK family to act at the post-transcriptional level by regulating mRNA stability and protein translation of IRAK-M, as has previously been reported with other cytokines, transcription factors and cell surface receptors [58]. Alternatively, there could be an autocrine/paracrine loop between p38^MAPK^ and STAT3 [59] that would also contribute to the IRAK-M overexpression elicited by nicotine. Further experiments are needed to explore both proposals.

The finding that the three signaling molecules (JAK2, STAT3 and PI3K/Akt) contribute equally to nicotine-induced IRAK-M expression (Figure 3B) indicates that these molecules can be chained to each other or converge on a common signaling pathway leading to induction of IRAK-M. There are two main possible signaling cascades for connecting JAK2, STAT3 and PI3K, and our phosphorylation data using the specific inhibitor of the first signaling molecule in the cascade confirm that both are feasible (Figure 3D–E). Through the first cascade, nicotine stimulation of α7 nAChR directly activates JAK2, which in turn could phosphorylate both PI3K and STAT3, as has already been demonstrated conclusively in previous studies [16], [52] and is also supported by our results in MØ (Figure 3D); both activated signals would eventually, and independently, results in increased IRAK-M expression. In fact, it has been reported that PI3K activation by stimuli other than nicotine, such as adiponectin or LPS, result in IRAK-M overexpression in macrophages [29], [54], [60], [61]. Although there is no experimental evidence for the transcriptional activation of the IRAK-M gene by STAT3, it is worth mentioning that the ChIP analysis done by the ENCODE project [62] using the lymphoblastic cell line GM12878 shows STAT3 binding sites within the first intron of the IRAK-M gene to be coincident with DNase I hypersensitive clusters and histone H3K27ac marks, features related to active regulatory elements. Indeed, STAT3 activates the transcription of other negative inflammatory regulators, like SOCS-3, in response to α7 nAChR activation in mice peritoneal macrophages [16]. The second cascade would be a signaling pathway through PI3K, activated or not by JAK2, which could phosphorylate STAT3 via other non-receptor tyrosine kinases like BMX [63]. In fact, this new alliance between PI3K and STAT3 has recently been reported in rat nodose ganglion neurons [64] and in some human cancer cell lines [63]. Our data in Figure 3E indicated that this alliance also occurs in MØ stimulated with nicotine.

Once it had been established that nicotine induced overexpression of IRAK-M via α7 nAChRs in MØ, we next explored the biological relevance such an up-regulation could have in the anti-inflammatory effect of nicotine. Our results substantiate its importance, as can be deduced from the failure of nicotine to dampen the LPS-induced pro-inflammatory response (TNF-α) in MØ transfected with specific siRNAs for the IRAK-M gene but not in cells transfected with the negative siRNA control (Figure 4B). Selective knock-down of IRAK-M expression in siRNA-1/−2 transfected cultures was clearly evident in the Western blots, as opposed to the lack of effect on the expression in cells transfected with a negative siRNA control (Figure 4A).

When immune cells are first subjected to LPS exposure or undergo TLR activation following an infection: 1) they activate a rapid inflammatory response (increasing the production of TNF-α and other inflammatory mediators); and then, 2) they reprogram their activity and become refractory to further LPS challenge through the influence of various molecular determinants, like IRAK-M overexpression [23], [36]–[38]. This refractory state implies that, when these tolerant cells again come in contact with a subsequent LPS stimulus, they respond with a reduced TNF-α production and an increased IRAK-M expression compared to those generated during prior exposure to LPS. Given the above scenario, we found that nicotine exposure, through a mechanism that is similar to that produced by LPS, also leads MØ toward a tolerant state regarding further LPS challenges (Figure 5B–C). Thus, following preincubation with nicotine, LPS-activated MØ show enhanced IRAK-M induction and a reduced pro-inflammatory response (lower TNF-α levels) in relation to LPS-activated cells not subjected to prior nicotine exposure. This hyporesponsive state of the immune response in addition to the sustained anti-inflammatory effect secondary to IRAK-M overexpression elicited by nicotine, would help explain the known beneficial or negative effects of smoking in different clinical scenarios [29]. Thus, smoking is beneficial in ulcerative colitis, a characteristic inflammatory bowel disorder in which the vast majority of patients (90%) are non-smokers, while in those with established disease, smoking results in a less severe course and a lower likelihood of requiring colectomy [65]–[67]. Also, induction of IRAK-M in smokers may be beneficial to the host in early sepsis by limiting its progression to septic shock and subsequent organ failure [25], [34]. In contrast, there are other settings in which overexpression of IRAK-M might prevent appropriate host defense against infection; this could partially explain why smokers are several times more susceptible to community-acquired pneumonia or to periodontal tissue infection than nonsmokers [68]–[70].

In summary, the findings of our study reveal that induction of IRAK-M in human macrophages is an intracellular regulator operating as an effector of the anti-inflammatory action of nicotine downstream from α7 nAChRs. Furthermore, we also found that nicotine generates a hyporesponsive state to subsequent challenge in macrophages. Since both nicotine effects could eventually limit inappropriate immune activation, or, conversely, compromise the normal immune response, they should be taken into account in smokers experiencing different pathologies in which IRAK-M overexpression may be influential [29].
